# The Nicaraguan Pediatric Dengue Cohort Study: Incidence of Inapparent and Symptomatic Dengue Virus Infections, 2004–2010

**DOI:** 10.1371/journal.pntd.0002462

**Published:** 2013-09-26

**Authors:** Aubree Gordon, Guillermina Kuan, Juan Carlos Mercado, Lionel Gresh, William Avilés, Angel Balmaseda, Eva Harris

**Affiliations:** 1 Division of Epidemiology, School of Public Health, University of California, Berkeley, Berkeley, California, United States of America; 2 Division of Infectious Diseases and Vaccinology, School of Public Health, University of California, Berkeley, Berkeley, California, United States of America; 3 Centro de Salud Sócrates Flores Vivas, Ministry of Health, Managua, Nicaragua; 4 Laboratorio Nacional de Virología, Centro Nacional de Diagnóstico y Referencia, Ministry of Health, Managua, Nicaragua; 5 Sustainable Sciences Institute, Managua, Nicaragua; Yale University, United States of America

## Abstract

Dengue, caused by the four serotypes of dengue virus (DENV), is the most prevalent mosquito-borne viral disease of humans. To examine the incidence and transmission of dengue, the authors performed a prospective community-based cohort study in 5,545 children aged 2–14 years in Managua, Nicaragua, between 2004 and 2010. Children were provided with medical care through study physicians who systematically recorded medical consult data, and yearly blood samples were collected to evaluate DENV infection incidence. The incidence of dengue cases observed was 16.1 cases (range 3.4–43.5) per 1,000 person-years (95% CI: 14.5, 17.8), and a pattern of high dengue case incidence every other year was observed. The incidence of DENV infections was 90.2 infections (range 45.2–105.3) per 1,000 person-years (95% CI: 86.1, 94.5). The majority of DENV infections in young children (<6 years old) were primary (60%) and the majority of infections in older children (≥9 years of age) were secondary (82%), as expected. The incidence rate of second DENV infections (121.3 per 1,000 person-years; 95% CI: 102.7, 143.4) was significantly higher than the incidence rate of primary DENV infections (78.8 per 1,000 person-years; 95% CI: 73.2, 84.9). The rigorous analytic methodology used in this study, including incidence reporting in person-years, allows comparison across studies and across different infectious diseases. This study provides important information for understanding dengue epidemiology and informing dengue vaccine policy.

## Author Summary

Dengue is a major public health problem worldwide with 40 million cases annually. We conducted a large-scale prospective cohort study of dengue virus infection in children aged 2–14 years in Managua, Nicaragua. The observed rate of dengue cases was 16.1 cases per 1,000 persons per year. The observed rate of dengue virus infections was 90.2 infections per 1,000 persons per year. The observed rate of dengue virus infections is similar to the rates reported in Asian cohort studies, while the rate of dengue cases was lower than that observed in Asian cohort studies. The rate of dengue cases varied more than the rate of dengue virus infections, and a clear pattern of high dengue case incidence every other year was observed. The rigorous analytic methodology used in this study allows comparison of incidence of dengue virus infections and dengue cases across studies and across different infectious diseases. Our estimates of the burden of dengue in Nicaraguan children have significant policy implications for dengue vaccines as they become available.

## Introduction

Dengue is the most common mosquito-borne viral infection worldwide, causing an estimated 40 million cases and 500,000 hospitalizations annually [1]. Most infections with the four dengue virus serotypes (DENV1-4) occur in urban and semi-urban areas of tropical and sub-tropical countries, where DENV is transmitted by *Aedes aegypti* and *Ae. albopictus* mosquitoes [2]. DENV infection results in a spectrum of disease, ranging from inapparent infection (50–90% of infections) to classic dengue fever, a self-limiting acute illness with headache, retro-orbital pain, myalgia, arthralgia, rash, and at times hemorrhagic manifestations, to life-threatening syndromes characterized by vascular leakage, hemorrhage, and shock [3]. Exposure to one serotype of DENV provides lifelong immunity to that serotype, but does not confer lasting protection to the other three serotypes; in fact, prior infection with a different DENV serotype is the single greatest risk factor for development of severe disease [4].

The earliest reports of dengue in the Americas date back to the 18^th^ century, and dengue caused epidemics in North and South America throughout the 20^th^ century [5]. Improved socioeconomic status, waste and water management, and behavioral factors controlled dengue in the United States, and the Pan American *Ae. aegypti* eradication campaign in the 1950's and '60's greatly reduced DENV transmission in Latin America [5]. However, when the program ceased, *Ae. aegypti* mosquitoes and DENV soon returned. In the early 1970's, only DENV-2 was endemic throughout the Americas, with limited reports of DENV-3 activity [6]. In 1977, DENV-1 was introduced, followed by DENV-4 in 1981 and an Asian DENV-3 genotype in 1994 [7]. Currently, all four serotypes circulate throughout the continent.

The first reported dengue epidemic in Nicaragua occurred in 1985, when >17,000 cases and seven deaths were documented, caused by DENV-1 and DENV-2 [8]. Intensive vector control efforts resulted in no dengue outbreaks until the early 1990's, when yearly outbreaks of DENV-1, DENV-2 and DENV-4 were reported, followed by a large DENV-3 epidemic in 1994–5 [9], . Since then, all four DENV serotypes have circulated, but unlike Asia where they are hyperendemic, in Nicaragua one serotype typically dominates each season [11], [12], [13], [14], [15]. In our cohort, epidemics peak during the rainy season (especially August-January), although a low level of cases occur throughout the year [16]. DENV is the only flavivirus known to be circulating in humans in Nicaragua, and since yellow fever is not an endemic disease, the Nicaraguan population does not receive the yellow fever vaccine (A. Balmaseda, unpublished).

The Pediatric Dengue Cohort Study, an ongoing prospective cohort study of dengue in children in Managua, Nicaragua, was established in August 2004 to determine the incidence of DENV infection and dengue cases, characterize the clinical spectrum of disease, and study viral and immunological determinants of DENV infection outcome [17]. A report presenting data from the first four years of the study has been published previously [16]. This paper presents results from the first six years of the Pediatric Dengue Cohort Study.

## Materials and Methods

### Ethics statement

This study was conducted as a collaboration between the Nicaraguan Ministry of Health and the University of California, Berkeley. The study was approved by the Institutional Review Boards (IRBs) at the University of California, Berkeley, the Nicaraguan Ministry of Health, and the International Vaccine Initiative. Written consent was obtained from a parent or guardian, or if the guardian was illiterate, the consent form was read aloud in the presence of a witness and the guardian's thumbprint was obtained in lieu of a signature, as approved by the IRBs. Verbal assent was obtained from all children aged six years and older.

### Study population

The Nicaraguan Pediatric Dengue Cohort Study (PDCS) is an ongoing prospective cohort study in children two to fourteen years of age in Managua, Nicaragua. A detailed description of the study design, methods, and study population has been published previously [17]. Briefly, recruitment into the study began through house-to-house visits in August 2004. All children two to nine years old living within the study area were invited to participate. At enrollment, families agreed to bring their children to the study health center, Health Center Sócrates Flores Vivas (HCSFV), for medical care at the first sign of illness. Each year, an annual blood sample was collected in July/August to enable assessment of DENV infection. The initial consent covered three years of study participation; in 2007, the study was extended for an additional three years; in 2009, the study was extended for an additional year, and in 2011, for another three years. During the first three years of the study, children were eligible to participate until they reached the age of 12. From the fourth year onwards, the protocol was modified to extend eligibility to 14 years of age. The focus of this report is the first six years of the study. The cohort was sized such that even in years of relatively low DENV transmission, a minimum number of symptomatic cases would be identified.

### Follow-up and case identification

Participants are provided with free medical care 24 hours/day, 365 days/year through study physicians at the HCSFV. Children requiring hospitalization are transferred to the study hospital, the National Pediatric Reference Hospital (Hospital Infantil Manuel de Jesús Rivera) by study staff. At the HCSFV, children are examined by study physicians, and medical data is recorded systematically on study collection forms. Data is collected on approximately 80 variables including vital signs, temperature, musculoskeletal pain, respiratory symptoms, gastrointestinal symptoms, indicators of dehydration, and rashes and other skin anomalies. Upon medical examination, children are categorized into one of four categories according to their symptoms: suspected dengue case meeting the WHO case definition; undifferentiated febrile illness; febrile illness with defined non-dengue focus; and non-febrile illness or injury.

### Clinical and laboratory definitions

#### Suspected dengue case

A child presenting with a fever and two or more of the following symptoms: headache, myalgia, arthralgia, retroorbital pain, rash, hemorrhagic manifestations or leukopenia.

#### Undifferentiated febrile illness

A child presenting with an undifferentiated fever of undefined origin.

#### Acute dengue case

An ill child who tested positive for dengue as evidenced by: 1) detection of DENV RNA by RT-PCR [13], [18], 2) isolation of DENV in C6/36 cells [13], 3) seroconversion as determined by a DENV-specific immunoglobulin M (IgM) capture enzyme-linked immunosorbent assay (ELISA) using paired acute and convalescent sera [19], and/or 4) a ≥4-fold rise in total antibody titer between acute and convalescent sera as measured by Inhibition ELISA [20], [21], with titer determined using the Reed-Muench method [17], [22].

#### 1997 WHO case classification

Dengue cases were classified according to the 1997 WHO Case Classification [3]. *Dengue Fever (DF)*: A dengue case that does not meet the definition of DHF or DSS. *Dengue Hemorrhagic Fever (DHF)*: A dengue case with the presence of: 1) fever or a history of fever; 2) hemorrhagic manifestations; 3) thrombocytopenia (≤100,000 platelets/ml) and 4) evidence of plasma leakage. *Dengue Shock Syndrome (DSS)*: DHF with evidence of circulatory failure consisting of: 1) hypotension for age or narrow pulse pressure (<20 mm Hg) and 2) clinical signs of shock (e.g., rapid weak pulse, cold clammy sign, poor capillary refill) [23].

#### 2009 WHO case classification

These criteria [24] were applied retrospectively to dengue cases that occurred in the cohort before the criteria were published. *Dengue with Warning Signs*: dengue cases with abdominal pain, persistent vomiting, fluid accumulation, lethargy, mucosal bleeding, fluid accumulation, liver enlargement, or increasing hematocrit with decreasing platelets. *Severe Dengue.:* Dengue cases with severe bleeding, severe plasma leakage leading to shock or fluid accumulation with respiratory distress, or organ failure or involvement as evidenced by liver ALT or AST ≥1,000, impaired consciousness, failure of the heart or other organs.

#### Inapparent DENV infection

A child whose paired annual serum samples demonstrated seroconversion (a titer of <1∶10 to ≥1∶10) or a ≥4-fold rise in antibody titer as determined by Inhibition ELISA and who did not experience a symptomatic DENV infection during the intervening year. Paired annual samples were run on the same ELISA plate to allow for a more accurate comparison of titers. The Inhibition ELISA has been previously evaluated in Nicaragua against the Hemagglutinin Inhibition assay [20], [21]. This evaluation showed a high concordance for seropositivity (sensitivity and specificity of the Inhibition ELISA of 98.9% and 100%, respectively) and a strong correlation of titer (Pearson's r = 0.80) between the two techniques. Moreover, to assess the reproducibility of the Inhibition ELISA assay, a subset of paired annual samples was run twice (3,510 samples corresponding to 1,755 paired annual samples). The agreement on infection outcome between the two runs was 97.9%, and the Kappa statistic was 0.836 (95% confidence interval (CI): 0.784, 0.889). Finally, the sensitivity of the Inhibition ELISA to capture DENV infections using annual samples was estimated by calculating the percentage of confirmed dengue cases that experience either seroconversion or a ≥4-fold rise in antibody titer. Of 325 dengue cases with available pre- and post-infection annual samples, 259 met the definition of a DENV infection using Inhibition ELISA; thus, the sensitivity of the assay was estimated to be 79.7% (95% CI: 74.8, 83.8). The sensitivity of the Inhibition ELISA was similar in DF cases when compared to DHF/DSS (data not shown).

#### Primary and secondary DENV infection

A DENV infection was classified as a primary DENV infection if seroconversion was observed and was considered a secondary DENV infection if a ≥4-fold increase in antibody titer was observed in paired consecutive annual samples, as determined by Inhibition ELISA [17]. If a child's serum contained anti-DENV antibody at enrollment or the child experienced a previous DENV infection during the cohort, prior to a documented subsequent DENV infection, this was also considered a secondary DENV infection. First, second, and third DENV infections were identified by counting the number of the infections documented in a participant who entered the cohort dengue-naïve, using a ≥4-fold rise in titer by Inhibition ELISA in paired annual samples to identify inapparent infections or diagnostic assays in acute and convalescent samples for symptomatic cases. The sensitivity of the Inhibition ELISA to capture primary DENV infections was estimated by calculating the seroconversion percentage in annual samples of primary dengue cases. Of 141 primary dengue cases, 125 seroconverted (sensitivity: 88.7%; 95% CI: 81.9, 93.2). Similarly, the sensitivity of the Inhibition ELISA to capture secondary DENV infections was estimated using secondary dengue cases that had demonstrated a ≥4-fold rise in titer (n = 184; sensitivity: 72.8%; 65.7, 79.0).

#### Dengue-naïve

A child who did not have detectable anti-DENV antibody at enrollment as evidenced by Inhibition ELISA and did not experience a DENV infection during the cohort study.

#### Non-dengue-naïve

A child who had anti-DENV antibody at enrollment as evidenced by Inhibition ELISA or who experienced a documented DENV infection during the cohort study.

### Statistical analysis

Follow-up time was calculated as the amount of time between enrollment and the end of the reported study period (June 30, 2010) or withdrawal from the study. For those lost to follow-up, person-years were calculated as the time between enrollment and the last contact with study personnel, plus one-half the time between the last contact and the date recorded as lost to follow-up. Dengue cases were excluded from contributing person-time for one month following illness. Analysis of DENV infections was limited to those participants who completed the year and contributed a blood sample at the beginning and the end of the year. Analysis of second and third DENV infections was further limited to participants who entered into the cohort dengue-naïve. In order to provide conservative estimates of DENV infection incidence, since the exact timing of the DENV infection could not always be ascertained, persons who experienced a DENV infection in a given year contributed person-time for that entire year. A Poisson distribution was used to calculate 95% CIs for the incidence rates. Participant age used in the analysis of dengue case incidence was defined on a weekly basis since their exact age at the time that they were a dengue case can be ascertained. For the DENV infection analyses, participant age was defined as the age of the child when their annual sample was collected. In order to adjust for the sensitivity of the Inhibition ELISA test, each child's study ID was randomly sampled with replacement to match the observed number of children in the dataset, and then all observations from the selected children were used to create a new dataset. The expected number of DENV infections was equal to the number of observed infections in this new dataset divided by the sensitivity of the Inhibition ELISA test. The sensitivity was obtained by determining the number of symptomatic DENV infections reported in this study that were correctly identified as DENV infections by Inhibition ELISA in paired annual samples. The incidence per 1,000 person-years was 1,000 times the number of DENV infections divided by the number of person-years in the new dataset ( = 1,000*infections/(sum(days)/365.25)). This was performed 1,000 times to calculate a mean incidence and confidence intervals. Statistical analyses were performed in STATA, version 12 (StataCorp LP, College Station, TX).

## Results

### Study participation

**Figure 1:**
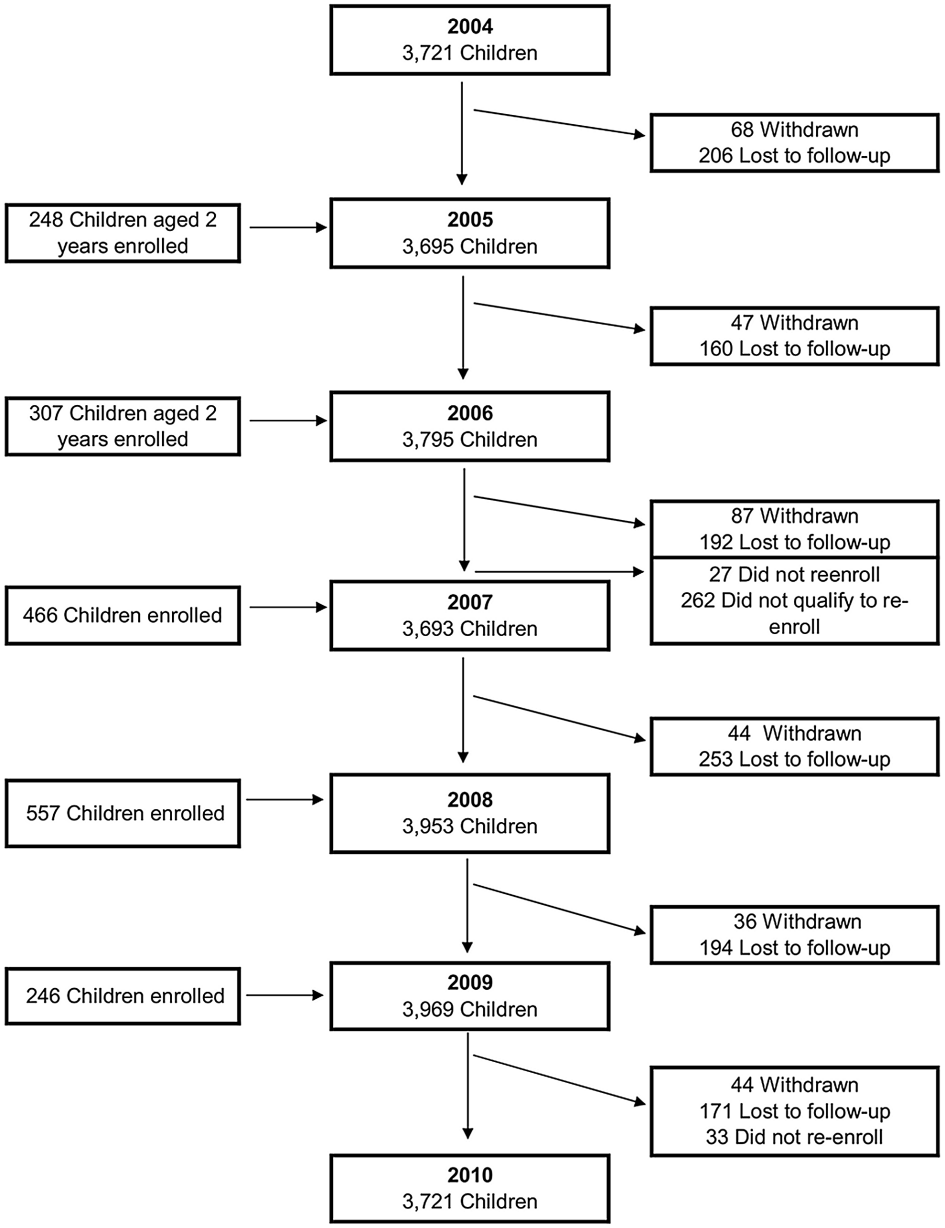
Flowchart of participants in the Pediatric Dengue Cohort Study, Managua, Nicaragua, 2004–2010.

During the first six years of the PDCS (August 2004 to June 2010), 5,545 children participated, contributing 21,839 person-years. Yearly participation ranged from 3,693 to 3,953 children (Table 1), and mean participation time was 3.9 years per child (range 0.02–5.9). A total of 1,204 (21.7%) children were lost to follow-up, 341 (6.1%) children completed the time that they had consented to participate in the study and were not re-enrolled into the study, and 340 (6.1%) were withdrawn (Figure 1). Of those lost to follow-up, 844 (62.2%) had moved and could not be contacted. Annual loss to follow-up ranged from 3.8% to 6.9%. Of the 341 children who did not re-enroll in the study, 278 (81.5%) did not qualify to re-enroll either because they had reached the maximum age for participation or had moved from the study area.

**Table 1:** Participant characteristics by year, Managua, Nicaragua, 2004–2010.

	2004–2005	2005–2006	2006–2007	2007–2008	2008–2009	2009–2010
	n = 3,721(%)	n = 3,695(%)	n = 3,795(%)	n = 3,693(%)	n = 3,953(%)	n = 3,969(%)
By sex						
Female	1,827 (49.1)	1,820 (49.3)	1,870 (49.3)	1,829 (49.5)	1,965 (49.7)	1,969 (49.6)
Male	1,894 (50.9)	1,875 (50.7)	1,925 (50.7)	1,864 (50.5)	1,988 (50.3)	2,000 (50.4)
By age (years)						
2–5	1980 (53.2)	1635 (44.2)	1378 (36.3)	1302 (35.3)	1288 (32.6)	1103 (27.8)
6–8	1360 (36.5)	1299 (35.2)	1297 (34.2)	1231 (33.3)	1254 (31.7)	1132 (28.5)
9–14	381 (20.6)	761 (20.6)	1120 (29.5)	1160 (31.4)	1411 (35.7)	1734 (42.7)

The number of participants from 2004–2005 to 2007–2008 has been previously reported [16].

### Dengue cases and disease severity

**Figure 2:**
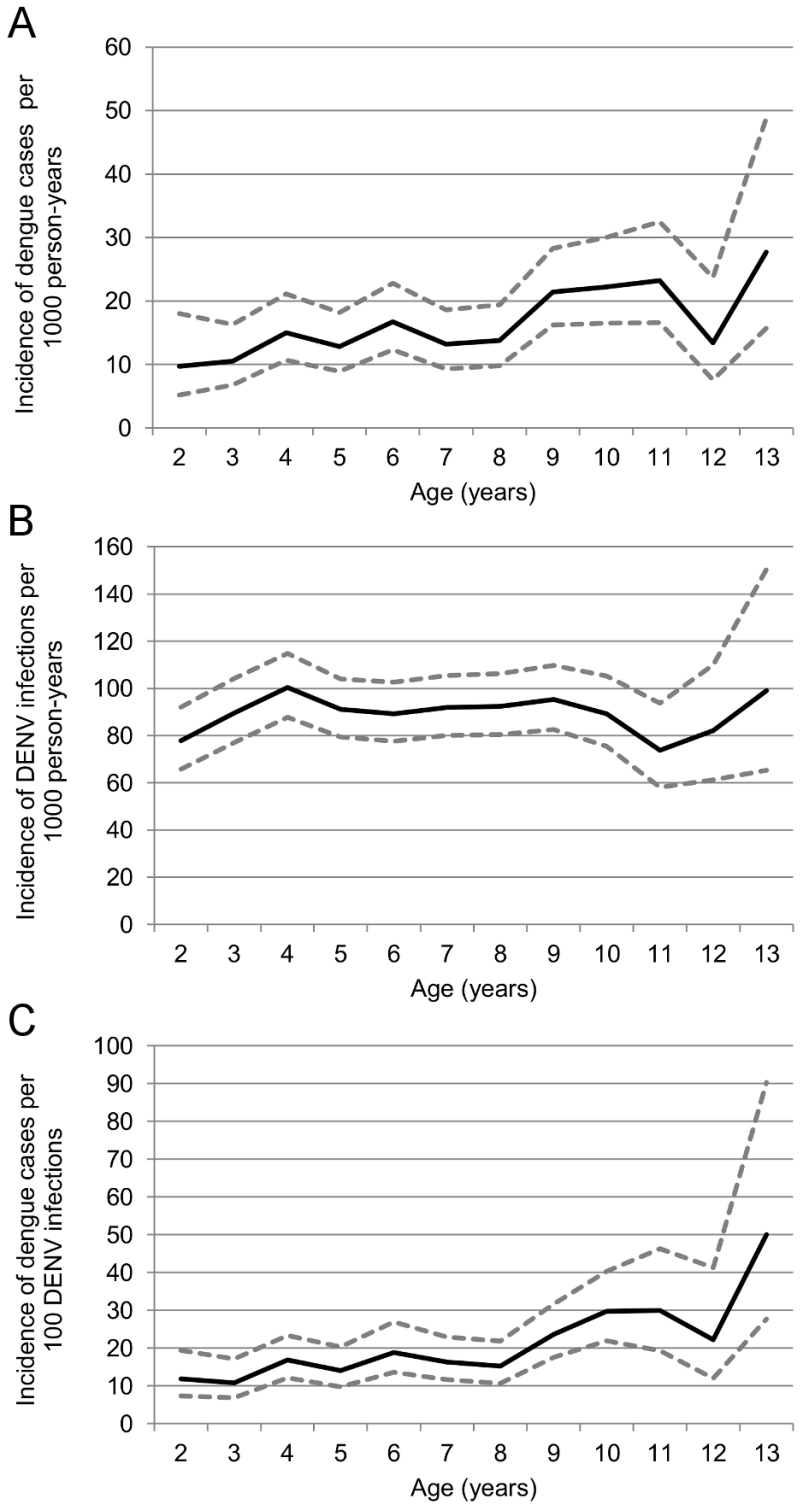
Incidence of DENV infection outcomes by age. A. Dengue cases per 1,000 person-years at risk. B. DENV infections per 1,000 person-years at risk C. Dengue cases per 100 DENV infections.

**Figure 3:**
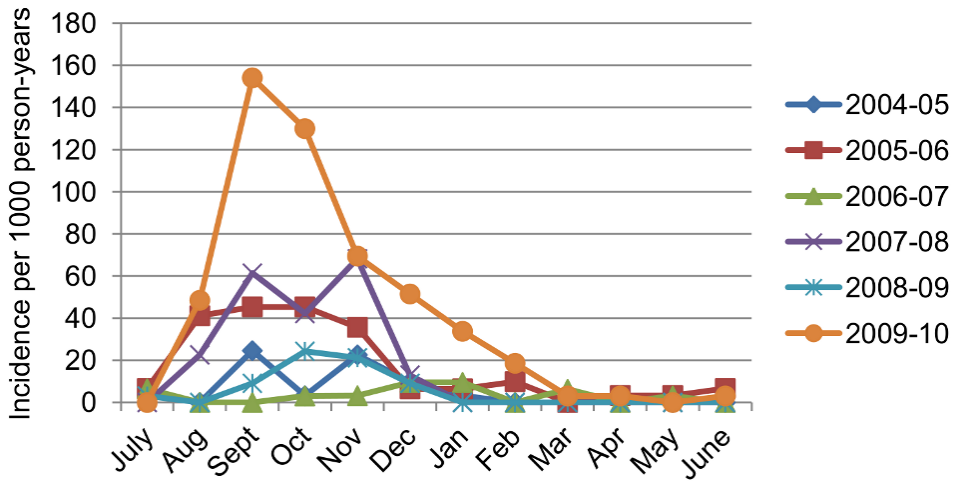
Incidence of dengue cases in the cohort by study year and month, August, 2004–June, 2010. The number of dengue cases by month from 2004–2005 to 2007–2008 has been previously reported [16].

In Years 1–6 of the PDCS, 2,601 suspected dengue cases or undifferentiated febrile illnesses were documented, of which 351 (13.5%) were laboratory-confirmed as dengue-positive, and 138 (39.3%) children with DENV infection were hospitalized. The overall incidence rate of dengue in the cohort was 16.1 dengue cases per 1,000 person-years (95% CI: 14.5, 17.8), with yearly symptomatic incidence ranging from 3.4 to 43.5 dengue cases per 1,000 person-years. The highest incidence of dengue was observed in children ≥8 years old (Table 2, Figure 2A). A majority of cases occurred from August to February, although limited numbers of dengue cases were observed year-round (Figure 3).

**Table 2:** Incidence of dengue cases, Managua, Nicaragua, 2004–2010.

	Person-years at risk	Dengue cases	Incidence per 1,000 person-years	95% CI
All participants	21,839.3	351	16.1	14.5, 17.8
By year				
2004–2005	2,942.8	17	5.8	3.6, 9.3
2005–2006	3,662.3	65	17.7	13.9, 22.6
2006–2007	3,783.7	13	3.4	2.0, 5.9
2007–2008	3,654.4	64	17.5	13.7, 22.4
2008–2009	3,887.7	22	5.7	3.7, 8.6
2009–2010	3,908.5	170	43.5	37.4, 50.6
By sex				
Male	11,052.0	180	16.3	14.1, 18.8
Female	10,787.3	171	15.9	13.6, 18.4
By Age (years)				
2–5	7484.6	93	12.4	10.1, 15.2
6–8	7211.3	105	14.6	12.0, 17.6
9–14	7143.4	153	21.4	18.2, 25.1

The number of dengue cases from 2004–2005 to 2007–2008 has been previously reported [16].

**Figure 4:**
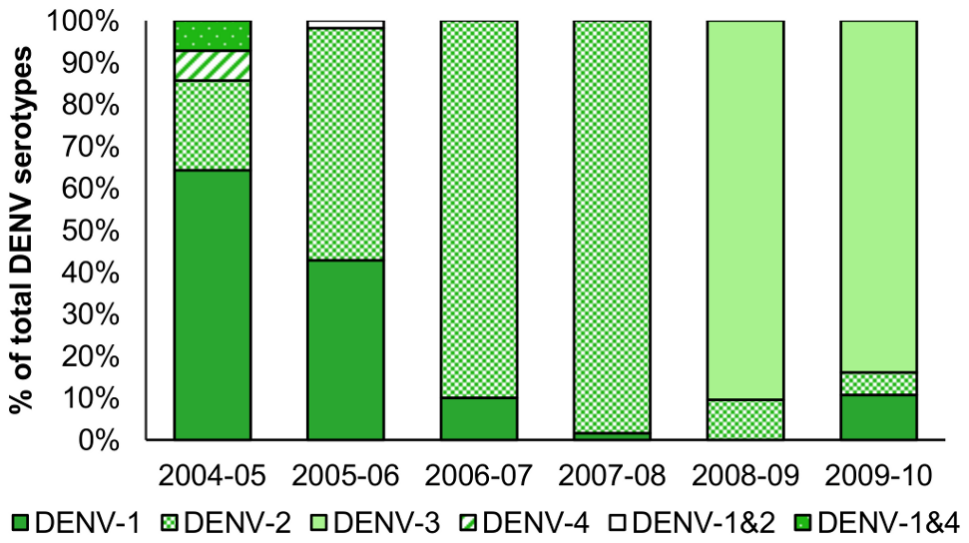
Distribution of DENV serotypes in dengue cases by study year. The percent of dengue cases infected by each DENV serotype, of the total infections with known serotype, are shown. Three hundred and thirty-one (94%) of the 351 dengue cases had serotype information available. Two cases of co-infection were detected: one DENV-1 & DENV-4 co-infection in 2004–05 and one DENV-1 & DENV-2 co-infection in 2005–06. The distribution of DENV serotypes in dengue cases from 2004–2005 to 2007–2008 has been previously reported [16].

In the first year of the study, a majority of symptomatic cases were caused by DENV-1 infection (52.9%); however, the percentage of cases caused by DENV-2 increased in 2005 through 2008 (study Years 2–4), reaching 95.3% of detected cases in 2007–2008 (Year 4) (Figure 4). DENV-3 entered the study population in 2008–2009 and was the predominant serotype in 2008–2009 (86.4%) and 2009–2010 (82.4%). Two cases of co-infection with two different DENV serotypes were also identified: a DENV-1 and DENV-4 co-infection in 2004–2005 and a DENV-1 and DENV-2 co-infection in 2005–2006 (Figure 4). A spatio-temporal video of dengue cases in the PDCS from 2004–2010 can be viewed at http://youtu.be/yQOEdsMhoSk.

Using the 1997 WHO definitions, of 351 dengue-positive cases, 319 (90.9%) had classic dengue fever, 21 (6.0%) had DHF, and 11 (3.1%) had DSS. The majority of these DHF/DSS cases occurred in two years of the study, with five DHF cases (23.8%) and three DSS cases (27.3%) in the 2007–2008 dengue season, and 12 DHF cases (57.1%) and seven DSS cases (63.6%) in the 2009–2010 dengue season. Using the 2009 WHO definitions of dengue severity, 182 cases were classified as dengue with warning signs (51.6% of dengue cases) and 53 (15.0%) as severe dengue. There was one death due to DENV infection during the six years of the cohort.

### DENV infections

Analysis of DENV infections was limited to participants who completed each year and contributed a blood sample at the beginning and at the end of the year. In total, 5,073 children contributed 19,708 person-years with 1,778 DENV infections, for an incidence rate of 90.2 infections per 1,000 person-years (95% CI: 86.1, 94.5) (Table 3). The incidence of DENV infections by study year ranged from 67.0 to 119.7 infections per 1,000 person-years. Excluding 14-year-old children due to small sample size, among one-year age groups, the highest DENV infection incidence was observed in four-year old children, with an incidence of 100.4 infections per 1,000 person-years, although the incidence was fairly constant across age groups (Figure 2B). Of the 5,073 children who contributed at least one set of paired samples, 3,570 (70.4%) did not experience a DENV infection during the study, 1,250 (24.6%) experienced one documented DENV infection, 231 (4.5%) experienced two DENV infections, and 22 (0.4%) experienced three DENV infections, according to serological analysis.

**Table 3:** Incidence of DENV infections, Managua, Nicaragua, 2004–2010.

	Person-years at risk	DENV infections	Incidence per 1,000 person-years	95% CI
All participants	19,708.3	1,778	90.2	86.1, 94.5
By year				
2004–2005	2,829.4	293	103.6	92.4, 116.1
2005–2006	3,426.6	410	119.7	108.6, 131.8
2006–2007	3,156.6	223	70.6	62.0, 80.6
2007–2008	3,341.9	240	71.8	63.3, 81.5
2008–2009	3,566.9	239	67.0	59.0, 76.1
2009–2010	3,386.8	373	110.1	99.5, 121.9
By sex				
Male	9,969.6	930	93.3	87.4, 99.5
Female	9,738.6	848	87.1	81.4, 93.1
By age				
2–5	7922.1	716	90.4	84.0, 97.2
6–8	6545.9	597	91.2	84.2, 98.8
9–14	5240.2	465	88.7	81.0, 97.2
By infection number				
1^st^	8,880.7	700	78.8	73.2, 84.9
2^nd^	1,137.3	138	121.3	102.7, 143.4
3^rd^	188.3	16	84.9	52.0, 138.7

The number of DENV infections from 2004–2005 to 2007–2008 has been previously reported [16]. The number of inapparent DENV infections in this paper vary slightly from our previous report due to the implementation of new quality control procedures which resulted in several revisions to the inapparent infection data.

**Figure 5:**
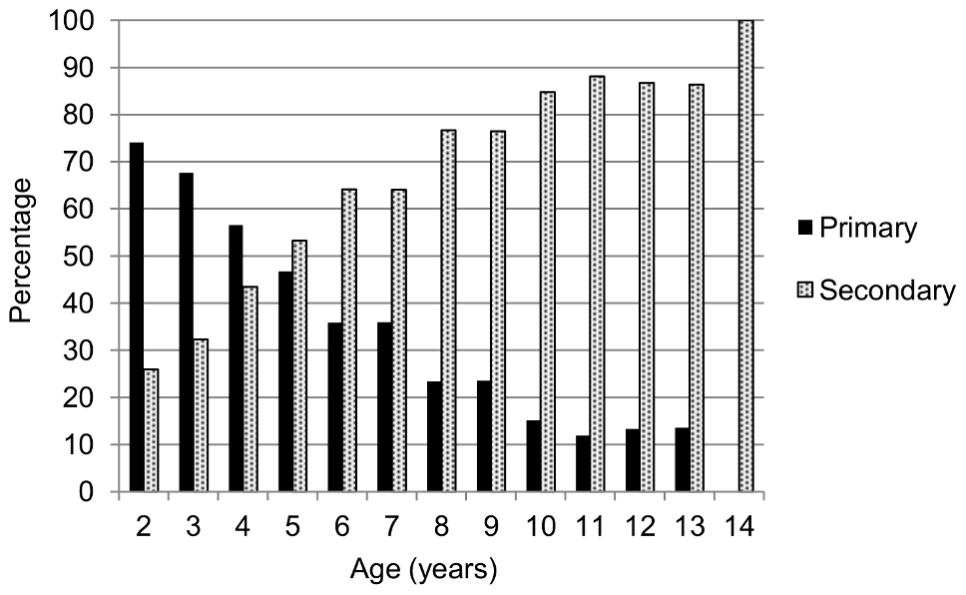
Percentage of primary and secondary DENV infections in the cohort by year of age. The percentage of primary and secondary immune responses, by age, from 2004–2005 to 2007–2008 has been previously reported [16].

Over the six years, 2,779 dengue-naïve children contributed 8,881 person-years of time. The incidence of primary DENV infections was 78.8 infections per 1,000 person-years (95% CI: 73.1, 84.9) (Table 4). Yearly incidence rates ranged from 45.2 to 105.3 primary DENV infections per 1,000 person-years. Excluding 12- and 13-year-old children due to small sample size, the highest incidence rate of primary DENV infection was observed in nine-year old children (109.4; 95% CI: 81.7, 146.6). Although the highest incidence rates of primary infections were seen in older children, relatively few older children were at risk for a primary infection and therefore the majority of DENV infections in older children were secondary (Figure 5).

**Table 4:** Incidence of primary and secondary DENV infections, Managua, Nicaragua, 2004–2010.

	Primary DENV Infections				Secondary DENV Infections			
	Person-years at risk	DENV infections	Incidence per 1,000 person-years	95% CI	Person-years at risk	DENV infections	Incidence per 1,000 person-years	95% CI
All participants	8,880.7	700	78.8	73.2, 84.9	10,829.6	1,078	99.5	93.8, 105.7
By year								
2004–2005	1,162.1	98	84.3	69.2, 102.8	1,667.3	195	117.0	101.6, 134.6
2005–2006	1,408.3	148	105.1	89.5, 123.5	2,018.3	262	129.8	115.0, 146.5
2006–2007	1,382.6	80	57.9	46.5, 72.0	1,774.0	143	80.6	68.4, 95.0
2007–2008	1,547.7	123	79.5	66.6, 94.8	1,794.2	117	65.2	54.4, 78.2
2008–2009	1,746.6	79	45.2	36.3, 56.4	1,821.4	157	87.8	75.2, 102.6
2009–2010	1,633.4	172	105.3	90.7, 122.3	1,754.4	180	114.5	99.8, 131.6
By sex								
Male	4,460.7	352	78.9	71.1, 87.6	5,510.9	578	94.0	86.1, 102.6
Female	4,419.6	348	78.7	70.9, 87.4	5,318.7	500	104.9	96.7, 113.8
By age								
2–5	5673.7	427	75.3	68.4, 82.7	2248.5	288	128.1	114.1, 143.8
6–8	2409.2	190	78.9	68.4, 90.9	4138.6	408	98.6	89.5, 108.6
9–14	797.9	83	104.0	83.9, 129.0	4442.4	382	86.0	77.8, 95.1

The number of primary and secondary DENV infections from 2004–2005 to 2007–2008 has been previously reported [16].

The 2,813 non-dengue-naïve children contributed 10,830 person-years. The incidence of secondary DENV infections was 99.5 infections per 1,000 person-years (95% CI: 93.8, 105.7) (Table 4), with annual secondary infection incidence rates ranging from 59.6 to 115.2 infections per 1,000 person-years. The highest incidence of secondary infections was observed in children aged five and under. However, when the percentage of primary and secondary infections was examined by year of age (Figure 5), the majority of DENV infections in younger children were primary, presumably due to the relatively small percentage of young children at risk for a secondary infection.

To examine the potential effects of both waning antibody levels and the use of the Inhibition ELISA assay on our estimate of overall DENV infection incidence, we performed a sensitivity analysis by determining how many symptomatic DENV infections reported in this study were correctly identified as DENV infections by Inhibition ELISA in paired annual samples. This yielded a sensitivity of 79.7%, which we then applied to our observed estimate of incidence to arrive at an incidence rate of 112.5 DENV infections per 1,000 person-years (95% CI: 107.7, 117.4). Therefore, our observed incidence is a conservative estimate of the true incidence in the cohort.

### Repeat DENV infections

Of the 700 children who entered the cohort dengue-naïve and experienced a primary DENV infection, 138 went on to experience a second DENV infection. The 700 children contributed 1,137 person-years of time, yielding an incidence rate of 121.3 second DENV infections per 1,000 person-years (95% CI: 102.7, 143.4) (Table 3). Of the 138 children who contributed 188.3 person-years of time at risk, 16 experienced third DENV infections, for an incidence rate of 84.9 third DENV infections per 1,000 person-years (95% CI: 52.0, 138.7) (Table 3). There were no fourth DENV infections observed during the 8.0 person-years of time at risk for a fourth infection.

### Dengue cases among DENV infections

The overall rate of symptomatic cases among DENV infections was 18.2 dengue cases per 100 DENV infections (Table 5). This rate varied dramatically by year; from 4.9 cases per 100 infections (95% CI: 2.7, 8.9) in 2006–2007 to 40.8 cases per 100 infections (95% CI: 34.8, 47.8) in the 2009–2010 season. The lowest rates of symptomatic cases were seen in the youngest age groups, two- and three-year olds, with rates of 11.8 and 10.8 cases per 100 infections, respectively. The highest rates were observed in the older age groups, especially in children aged nine and over, with rates of 23.6 to 29.9.

**Table 5:** Incidence of dengue cases among DENV infections, Managua, Nicaragua, 2004–2010.

	DENV infections	Dengue cases	Incidence of cases per 100 DENV infections	95% CI
All participants	1,778	325	18.2	16.4, 20.4
By year				
2004–2005	293	17	5.8	3.6, 9.3
2005–2006	410	64	15.6	12.2, 19.9
2006–2007	223	11	4.9	2.7, 8.9
2007–2008	240	60	25.0	19.4, 32.2
2008–2009	239	21	8.8	5.7, 13.5
2009–2010	373	152	40.8	34.8, 47.8
By sex				
Male	930	166	17.8	16.1, 21.9
Female	848	159	18.9	15.3, 20.7
By age (years)				
2–5	716	98	13.7	11.2, 16.7
6–8	597	100	16.8	13.8, 20.4
9–14	465	127	27.3	23.0, 32.4

## Discussion

To date, this is one of the longest continuous, large-scale, community-based prospective cohort study to characterize dengue cases and DENV infections, and it is currently ongoing. We show that there is a high incidence of DENV transmission in children in Managua, Nicaragua, leading to a substantial incidence of dengue cases, a large proportion of which were hospitalized. Although quite variable year-to-year, on average approximately six times as many DENV infections as cases were documented, illustrating that the number of symptomatic cases substantially underestimates DENV transmission in Nicaragua, consistent with our previous report on this cohort [16] and reports from other regions [16], [25], [26], [27].

Several prospective cohort studies of dengue and DENV infection in Asian and Latin American countries have been reported [16], [25], [27], [28], [29], [30], [31], [32], [33]. In Indonesia, during a one-year period of a study in children 4–9 years old, 23.2% of children were reported to have experienced a DENV infection [28]. In a seven-month dengue season during a prospective serological study in Thai schoolchildren aged 4–16 years, 5.6% of the children experienced a DENV infection, of whom 86% presented as asymptomatic or minimally symptomatic (defined as absent from school for one day) [29]. In a three-year prospective cohort study of DENV infections in Thai elementary schoolchildren, 5.8% of the children were infected with DENV, with 53% of infections presenting as inapparent DENV infections [25]. In a four-year prospective study of DENV infections in Thai children, 2.3% of children experienced a DENV infection per season, and the ratio of inapparent-to-symptomatic infection was 1.8∶1 [34]. In Iquitos, Peru, an incidence rate of 20–30 DENV-1 or DENV-2 infections per 1,000 person years was observed in a 3.5 year-long cohort study of ∼2,400 children and adults in years where the population had previously been exposed to DENV-1 or DENV-2; however, a peak rate of 890 infections per 1,000 person-years was observed with the introduction of DENV-3 into this population that was completely susceptible to DENV-3 [32]. The overall incidence of DENV infections (90.2 per 1,000 person-years) observed in this study in Nicaragua was within the range of the estimates reported in studies in Asia, while the incidence of dengue cases (16.1 per 1,000 person-years) was in general lower than observed in Asia. The incidence rate of second DENV infections in the Nicaraguan PDCS was significantly higher than that of primary infections, presumably due to the fact that children who have already experienced a primary DENV infection and are at risk for second DENV infections live in areas where they are more likely to be exposed to DENV than the overall dengue-naïve study population, who are at risk for primary DENV infections. The rates of second and third DENV infections were not significantly different.

In this manuscript, two dengue classification schemes, from the 1997 and the 2009 WHO Guidelines, were used [3], [24]. This allows for comparison to historical as well as future studies. The percentage of dengue cases qualifying as severe dengue (15%) or dengue with signs of alarm (53%) was higher than those meeting the traditional DHF/DSS classification (9%), consistent with other reports [35]. Overall, the observed rate of dengue cases among DENV infections was 18.2 cases per 100 infections (range 4.8–40.8 cases per 100 DENV infections), and a clear pattern of alternating seasons of high and low dengue case incidence was observed. This pattern was not due to differences in DENV infection rates, but rather to differences in the rate of dengue cases per DENV infection. As reported in the first four years of this study [16] and contrary to what has been described in Thailand [25], [26], [27], no positive correlation between the incidence of DENV infections and the incidence of dengue cases among DENV infections by year was observed. There were two years in particular where the rate of dengue cases per 100 DENV infections was high, the 2007–2008 dengue season (25.0 infections per 100 person-years) and the 2009–2010 dengue season (40.8 cases per 100 DENV infections). An increase in disease severity was also observed in the same two years [15], [36]. In 2007–2008, several factors appear to have contributed: waning DENV-1 immunity followed by DENV-2 resulted in more disease severity after a period of approximately two years, plus a clade replacement in DENV-2 led to greater disease severity in DENV-3-immune children infected by the replacing DENV-2 clade, NI-2B [15]. In 2009–2010, an unusual overlap with influenza pandemic strain H1N1 may have led to the observed atypical dengue presentation, more symptomatic DENV infections, and greater disease severity [36].

In this study, the highest incidence of primary infections was observed in older dengue-naïve children. This seems counter-intuitive, as the majority of DENV infections in older children are secondary (Figure 5). However, one possible reason for the higher rates of primary infections in older dengue-naïve children is that older children spend more time away from the house and thus have more opportunity to encounter DENV-infected mosquitoes both in the house and in other venues. Interestingly, although the majority of secondary DENV infections are in older children, the highest incidence rates of secondary infections were observed in the youngest children. Two possible explanations for this are first, that older non-dengue-naïve children may have already experienced more than one previous DENV infection and therefore have increased immunity (type-specific immunity to more serotypes and presumably some level of cross-reactive immunity), whereas younger non-dengue-naïve children are more likely to have only experienced a single prior DENV infection and are therefore more likely to be susceptible to the circulating DENV serotype. In fact, some of the older children may have experienced infection with all four DENV serotypes and therefore may not be at risk for infection. However, since many children enter the cohort non-naïve, we are unable to determine who is no longer at risk and therefore should not contribute person-time. Another possible explanation is that there may be a small percentage of the general population that is at greatest risk for DENV infection due to environmental or behavioral risk factors and therefore some children may experience multiple infections at an early age, thereby increasing the rate of secondary infection in some younger children. These findings highlight the importance of analyzing infection incidence rather than only proportions.

The strengths of this study include its large size, low loss to follow-up, high participation rates, and year-round follow-up. Many dengue cohort studies only operate during the dengue season, which results in an underestimate of annual incidence of dengue. The rigorous analysis used here, including incidence reporting in person-years, allows comparison across studies and across different infectious diseases. We are currently investigating factors that affect the incidence of symptomatic cases among DENV infections. Another strength of the study is the extensive use of information technologies and intensive quality control procedures since the study's inception [17].

Limitations of the study include ascertainment of cases through enhanced passive surveillance. Thus, some dengue cases may not have been detected due to parents not bringing their child in for healthcare [17]. Another limitation is that serum samples for cohort-wide serological testing were only available from participants once per year; however, due to the longevity of the study, more frequent sampling is not tolerated well by cohort participants. In this study, a ≥4-fold rise in antibody titer in paired annual samples was used to define a DENV infection. It is known that antibody levels begin to wane several months post-infection in dengue cases, but little to no information is available about antibody kinetics in inapparent DENV infections. A third limitation is that our annual sample was collected in July/August of each year, and during several of the study years, dengue transmission began during the sampling period. Thus, it is possible that some individuals may have been sampled very close to a DENV infection. This would result in misclassification of their infection by study year or classification as a DENV infection both in both years (in those individuals whose antibody levels were still rising at the time of sampling) when in fact they only experienced one DENV infection. Additionally, we exclude acute dengue cases for one month following infection, which makes incidence estimates conservative as children are likely protected from re-infection for substantially longer. This study relies on Inhibition ELISA to assess inapparent infections. Although no gold standard formally exists for assessing inapparent DENV infection, the plaque reduction neutralization test (PRNT) is considered the best method. However, PRNT is extremely labor-intensive, and given the resources available for the study, PRNT or other flow-based neutralization assays [37], [38] were not a feasible laboratory technique for a cohort of this size. Finally, as this study is limited to children, we are unable to provide information on the burden of dengue in adults in Nicaragua; however, establishing the incidence of dengue in children is particularly important as it is expected that they will be the first target population once there is a licensed dengue vaccine.

In summary, we have reported substantial incidence of DENV infections and dengue cases in children in Nicaragua using rigorous analytic methods. We have included several additional years of the cohort study and have presented the data in a form that can be readily used for comparison with incidence rates for other studies or other diseases and extrapolation for burden of disease estimates. Although costly and labor-intensive, cohort studies are the only way to determine the incidence of DENV infection and one of the few ways to capture the true rate of disease, which is greatly underestimated via national passive surveillance systems [39], [40]. These data are then utilized as a benchmark for establishing expansion factors that are used to estimate the actual disease burden [39], [41], [42] and the economic cost of the disease [43], [44], [45], as well as for cost-effectiveness studies of vaccines or other interventions [46], [47]. Thus, our incidence estimates have significant policy implications for dengue vaccines as they become available.

## Supporting Information

Checklist S1 **STROBE Checklist for cohort studies.**
(DOCX)
